# Small-Pox and the Public Schools of Atlanta

**Published:** 1882-07

**Authors:** W. F. Slaton

**Affiliations:** Superintendent Schools


### SMALL-POX AND THE PUBLIC SCHOOLS OF ATLANTA.

OFFICE SUP'T PUBLIC SCHOOLS,
ATLANTA, GA., June 20, 1882.

DR. JAMES B. BAIRD, *Secretary of the Board of Health:*

DEAR SIR—Nearly two years ago, I received from you, as Secretary of the Board of Health of the city of Atlanta, a communication calling my attention to the importance of the enforcement of the rule requiring the vaccination of all children attending the public schools; and believing that some good may result to other communities from our experience, I place at your disposal a few facts.

At the time the note of warning was sounded, there were in actual attendance in the public schools of Atlanta about thirty-six hundred children, twenty-eight hundred and seventy-five of whom had never been vaccinated. Your letter, together with other communications from superintendents of schools in Northern cities, was laid before the Board of Education at its first meeting, and I was directed to enforce the law to the letter. It was an easy matter to prevent the entrance of other children into the schools until the law was complied with, but what to do with the thousands who were already in, and who had not complied, presented a serious question. The excellent management, however, of a very sensible committee on sanitary affairs prevailed over all obstacles, and on the 1st day of December last I had in my office a certificate of vaccination, signed by Dr. Roach, chairman of the committee, or by Dr. Powell, the other practicing physician of the committee, for every child—*white* or *colored*—in the public schools. It is due the ward physicians to say, that much of the work was done by them, by order of the mayor and city council. It is proper here also to state that the Board did not dictate what physician should vaccinate the children—any one might do that—but Drs. Roach and Powell were held responsible for its success, and no child was admitted until one or the other of these physicians certified to the fact. When the small-pox made its first appearance in the city, there were forty-two hundred and fifty children attending the schools. There were two white children in attendance who came from a family in which a young man died from small-pox. There were others who lived on an adjoining lot where another death occurred. The colored people, not believing the disease to be small-pox, appeared at divers times on the streets with the disease in the stage of eruption before they were discovered by the active health officers, thereby exposing the children in many portions of the city to the dread disease. Nor is this all. Houston street colored school, numbering over five hundred children, is separated from what is called the Cameron block by a plank fence only. In this block three cases of small-pox occurred. From the corner of Butler and Houston streets, not a stone's throw from the school, was taken another case of the disease. Twenty-one children (colored) belonging to the school, from six different families, in which there were seven deaths from small-pox, were under quarantine for three weeks. *Not one child, white or colored, belonging to the public schools of Atlanta, has had small-pox, nor has a single child had even varioloid.*

These facts speak for themselves. Too much praise cannot be given to the Board of Education for enforcing the law in the face of what at one time threatened to be a storm of opposition. The skill of our committee physicians as well as their patience through nineteen months of labor, should not be forgotten by the parents of these children, many of whom doubtless owe to them their lives.

In all this work Mayor English has stood firm as a rock, and both by his personal influence and official authority, has protected the city which honors him as its worthy chief.

And now, sir, but a word more. I am no physician—I lay no claim to medical knowledge, but I have seen the small-pox scourge twice before, once when a child, and once in the late war, and I venture the assertion that but for the prompt, firm stand taken, first, by the Board of Education, and afterwards by the city authorities, Atlanta would to-day have been a charnel house for the dead.

The schools have not lost a day on account of the presence of small-pox in the city, and were never in a more flourishing condition than at the present time.

Doctors may differ as to unsettled theories, but when facts such as these occur in our own midst, it is time to end the discussion regarding the utility of vaccination. Let other school boards and other cities profit by our experience. Very respectfully, W. F. SLATON,
Superintendent Schools.

